# Designing an ethnographic interview for evaluation of micronutrient powder trial: Challenges and opportunities for implementation science

**DOI:** 10.1111/mcn.12804

**Published:** 2019-10-17

**Authors:** Courtney H. Schnefke, Alison Tumilowicz, Gretel H. Pelto, Seifu Hagos Gebreyesus, Wendy Gonzalez, Mélanie Hrabar, Shanzeh Mahmood, Catia Pedro, Melanie Picolo, Edna Possolo, Oana A. Scarlatescu, Dessie Tarlton, Julia Vettersand

**Affiliations:** ^1^ Public Health Research Division RTI International Research Triangle Park North Carolina; ^2^ Global Alliance for Improved Nutrition (GAIN) Geneva Switzerland; ^3^ Division of Nutritional Sciences Cornell University Ithaca New York; ^4^ Department of Reproductive Health and Health Service Management, School of Public Health Addis Ababa University Addis Ababa Ethiopia; ^5^ Independent Consultant Cape Town South Africa; ^6^ Maternal and Child Survival Program/PATH Maputo Mozambique; ^7^ Department of Nutrition Ministry of Health Maputo Mozambique; ^8^ Independent Consultant Maputo Mozambique

**Keywords:** communication challenges in multidisciplinary research, Ethiopia, focused ethnographic research, formative evaluation, implementation science in nutrition, micronutrient powders

## Abstract

The evidence base for micronutrient powder (MNP) interventions predominantly consists of quantitative studies focused on measuring coverage, utilization, and/or biological outcomes. We need other types of studies to broaden the scope of our knowledge about determinants of MNP programme effectiveness. Addressing this knowledge gap, this paper focuses on the process of designing an ethnographic research protocol to obtain caregivers' perspectives on the factors that influenced their use of intervention delivery services and their adherence to MNP recommendations. The research was undertaken within the context of formative evaluations conducted in Mozambique and Ethiopia. Ethnography provides a means for acquiring and interpreting this knowledge and is an approach particularly well suited for formative evaluation to understand the response of a population to new interventions and programme delivery processes. We describe decisions made and challenges encountered in developing the protocol, and their implications for advancing methodology in implementation research science. In addition to a core team of three investigators, we added an “advisory group” of 10 experts to advise us as we developed the protocol. The advisory group reviewed multiple drafts of the interview protocol and participated in mock interviews. In the protocol development process, we faced the issues and made decisions about concerned gaps in content, cultural adaptations and comprehension, and interview guide structure and format. Differences between the core team and the advisory group in methodological approaches to the structure and content of questions call attention to the importance of establishing greater communication among implementation scientists working in nutrition interventions.

**Key messages**

- Pretesting with an advisory group in the process of developing an ethnographic interview protocol for process evaluation played a vital role to improve structure and content of the protocol.
- A primary theme in the advisory group's feedback was criticism of the “conversational tone” of the open‐ended questions. A second, related concern was the balance between “broadness” and “specificity” of questions.
- Findings illustrate how fundamental features of ethnography are different from survey‐type research.
- Improvements to the protocol, along with results of using it described in accompanying papers of this supplement, demonstrate the value of involving an advisory group in implementation research.

## 1 INTRODUCTION

Although there is substantial evidence to support the selection of a micronutrient powder (MNP) intervention as part of an integrated strategy to address infant and child undernutrition in low‐ and middle‐income countries (World Health Organization, 2016), much remains to be learned about the challenges of realizing its potential (Dhillon et al., 2017). Adherence is widely recognized as critical for achieving impact in long‐term therapies in the treatment of disease (World Health Organization, 2003) and for sustained use of micronutrient supplements (Galloway & McGuire, 1994; Nagata, Gatti, & Barg, 2012; Reerink et al., 2017). When we implement MNP interventions, and nutrition programmes more broadly, we are usually introducing an intervention to make it possible for individuals, households, or communities to obtain better nutrition. Nutrition interventions are therefore always situated in a sociocultural context, and knowledge about that context is a fundamental prerequisite for adherence to recommendations and ultimately to their effectiveness.

To date, few studies have been conducted in the context of programme evaluations to elucidate factors that facilitate or impede caregivers' adherence to use recommendations for MNP. In a recent literature review of studies related to MNP adherence, including studies of children in the 6‐ to 59‐month age range, we found that only 12 of the 35 studies used qualitative methods, and seven of these were of short duration (2 days to 2 months; Tumilowicz, Schnefke, Neufeld, & Pelto, 2017). At present, the evidence base for MNP interventions predominantly consists of quantitative studies focused on measures of coverage, utilization of programmes, and/or biological outcomes. These studies, which use close‐ended questions and precoded answers, are based on issues of concern from the perspective of investigators or programme implementers. This approach yields valuable information but is insufficient. We also need information about utilization from the perspective of intended beneficiaries. This requires different methodological research techniques that permit us to examine not only the beneficiaries intervention experiences but also the factors that are influencing these experiences. Such data are essential for identifying barriers and facilitators to programme utilization and adherence. This paper focuses on the process of designing an ethnographic research protocol to obtain caregivers' perspectives on the factors that influenced their use of intervention delivery services and their adherence to MNP recommendations. The studies were undertaken within the context of a broader formative process evaluation.

Process evaluations that are undertaken for the purpose of “course correction” of an intervention programme (*formative use*) and/or to explain programme outcomes (*summative use*; Saunders, Evans, & Joshi, 2005) are becoming an integral aspect of nutrition implementation research (Board Members of the Society for Implementation Science in Nutrition, 2018). Although the maturity of evaluation research as a scientific discipline is reflected in public health and nutrition in multiple forms, including a large body of publications in scientific journals, text books, training programmes, and presentations in professional organizations and in established funding platforms, formative process evaluation (hereafter “formative evaluation”) is not yet systematically incorporated into nutrition programme improvement.

Regardless of the topic or motivation of the study, the central purpose of ethnography is to obtain the emic view—the insider's perspective (Dykes & Flacking, 2016; P. J. Pelto & Pelto, 1978; J. J. Schensul & LeCompte, 2016). The difference between ethnography and other social science methods of investigation is that ethnographers aim to discover what people do and why and then use what they learned to build theories, as contrasted with studies that are aimed at assessing the validity of existing theories, including theories of behavioural change. Ethnography provides a means for acquiring and interpreting this knowledge and is an approach particularly well suited for the purpose of formative evaluation when we need to understand the response of a population to new interventions and programme delivery processes (Fetterman, 1984; Patton, 2002).

In this paper, we draw on our experiences in two pilot projects that were undertaken to inform planning for scaling up the delivery of MNP in public health programmes in Ethiopia and Mozambique. Results of the evaluations are described in other papers in this supplement (G. H. Pelto et al., 2019; Tumilowicz et al., 2019; Tumilowicz et al., 2019). Here, we describe the decisions we made and the challenges we encountered in developing an ethnographic research protocol, as well as the implications of our experiences for advancing methodology in implementation research science. Our rationale for devoting a paper exclusively to methodological issues related to developing an ethnographic research protocol is that formative evaluation methodologies in nutrition are still in a relatively early stage of development, and empirical research papers on this topic will facilitate its further development.

## 2 METHODS

### 2.1 Theoretical and methodological background of the caregiver interview guide

Historically, many of the research activities that comprise formative evaluation were undertaken, both informally and formally, under various rubrics. Informally, the results are often found in the sections of evaluation reports labelled “lessons learned.” Formally, they have sometimes been characterized under the rubric of “operations research.” For example, an extensive research effort to assess a set of nutrition interventions in rural Haiti described this effort as “operations research” (Loechl et al., 2009). The recent focus on these critical processes has been accompanied by a shift in terminology, which explicitly embraces the concepts and practice of systematic process evaluation for formative and summative uses (Kim et al., 2015; Nguyen et al., 2014).

The formal use of qualitative research methods to evaluate nutrition programmes was proposed by Scrimshaw and Gleason (1992) and Scrimshaw and Hurtado (1987). In recognition of the need for providing guidance on how to conduct this type of research, the *Rapid Assessment Procedures* manual was developed by Scrimshaw and Hurtado and supported by training workshops (Scrimshaw & Hurtado, 1987). In subsequent years, there has been a trend to use mixed methods (Rawat et al., 2013). Currently, there are multiple definitions of “mixed methods,” but they share an emphasis on using both qualitative and quantitative methods. For example, in an early book describing this approach, Creswell (2003) define mixed methods as “a methodology for conducting research that involves collecting, analysing, and integrating (or mixing) quantitative and qualitative research (and data) in a single study or a longitudinal program of inquiry” (Creswell, 2003). In applied ethnographic research, there is a long tradition of mixed methods studies across a range of topics (J. J. Schensul & LeCompte, 2012), but as Pelto recently observed, some topical areas lend themselves more readily to a mixed methods approach than others (P. J. Pelto, 2017). He suggests that investigations of nutrition‐related behaviours are particularly well suited to combined qualitative–quantitative methods.

The formative evaluations of the MNP trials in Ethiopia and Mozambique consisted of two intersecting streams of inquiry: examination of programme outcomes and exploration of delivery experiences and MNP use from the perspectives of intended beneficiaries, that is, caregivers. For the latter, we selected a focused ethnographic approach because our previous experience with the focused ethnographic study for infant and young child feeding (G. H. Pelto & Armar‐Klemesu, 2014, 2015; G. H. Pelto, Armar‐Klemesu, Siekmann, & Schofield, 2013) suggested that this methodology could effectively elucidate the information that was required to answer the studies' specific objectives, which included (a) obtaining caregivers' perspectives on the factors that influenced their use of intervention delivery services and their adherence to MNP recommendations and (b) identifying factors that modified acceptance and utilization of MNP by caregivers and their children. Although focused ethnography is a mixed methods approach that typically involves qualitative and quantitative research techniques, we decided to use only in‐depth interviews and forego the application of quantitative cognitive mapping and other (e.g., social network mapping) techniques. This decision was made because we were undertaking a quantitative survey to measure programme outcomes, which would provide other types of data on acquisition and adherence behaviours to complement the in‐depth interview data, and we were concerned about the time demands on respondents of the interview. In our commitment to gathering emic data, our goal was to construct an in‐depth and open‐ended interview protocol that utilized qualitative interviewing principles that facilitated a full exploration of caregivers' experiences with programme delivery and use of MNP (Dykes & Flacking, 2016; S. L. Schensul, Schensul, & LeCompte, 1999).

### 2.2 Sequence of development of the caregiver interview guide

Developing the ethnographic caregiver interview protocol (hereafter “interview guide”) was organized into four phases:

Phase I Initial development of a draft of the caregiver interview guide by the core team
Phase II Critical review of the caregiver interview guide with a group of experts (hereafter, Project Advisory Group [PAG])
Phase III Core team revisions to the caregiver interview guide
Phase IV Pretesting, field testing, and cognitive testing

This paper covers Phases I–III. We do not include Phase IV or describe in detail the revisions that were necessary to adapt the guide for the different delivery systems that were used in Ethiopia and Mozambique. We made the decision to omit discussion of these topics to devote sufficient attention to a formative evaluation activity that we feel has received less attention in nutrition, namely, initial guide development. In our view, research guidelines on pretesting and field testing instruments are more systematized, more widely available, and more commonly utilized by implementation researchers, compared with initial development of interview tools (Collins, 2003).

### 2.3 Phase I: Initial development of the caregiver interview guide by the core team

Phase I was the responsibility of a small team, hereafter referred to as “the core team” (C. H. S., A. T., and G. P.). Two activities were used to develop the first draft of the interview guide.

1. A list of key topics and questions to address was generated based on a review of the literature on MNP interventions (Tumilowicz et al., 2017).
2. A review of the theoretical framework and the modules of the focused ethnographic study protocols for infant and young child feeding to identify question formats and determine what additional questions would be required to address MNP‐related topics (G. H. Pelto & Armar‐Klemesu, 2014).

The initial set of questions prepared by the core team included questions to explore caregivers' (a) interactions with the delivery system; (b) acceptance and utilization of MNP; (c) beliefs, perceptions, and behaviours related to MNP; (d) child food preparation practices; and (e) child feeding practices. In the two study sites, different questions were sometimes required because of the differences in the delivery systems in the two programmes: In Mozambique, the plan was for caregivers to receive vouchers from NGO or government health workers and redeem them for MNP sachets (locally branded *VitaMais*) at local shops. In Ethiopia, the MNP sachets (locally branded *Desta*) were distributed directly through government health extension workers.

After the initial draft of questions was prepared, the wording of questions was examined to ensure they accorded with good ethnographic interviewing techniques and to avoid imposing preconceived assumptions (Seidman, 2013; Spradley, 1979). At this early stage, we also recognized that the sequencing of questions and skip patterns would be challenging because some questions pertained only to specific categories of respondents. These categories were defined as caregivers who report currently using MNP (referred to in this paper as “continuing users”); caregivers who report having discontinued using (referred to as “noncontinuing users”); and for Mozambique where MNP was delivered through a voucher system, caregivers who report not redeeming vouchers (referred to as “nonredeemers”). Another reason for skip patterns in the interviews was that many of the follow‐up questions were based on respondents' prior responses. Consequently, the initial free‐flowing narrative of questions and skip pattern instructions in the first draft required attention to facilitate interviewers' work.

### 2.4 Phase II: Critical review of the caregiver interview guide with the PAG

After the core team completed a provisional draft of the interview guide, the next step was to seek advice from an informally constituted group of experts, hereafter referred to as the PAG (see Table 1.) We identified and selected 10 individuals, all of whom were already involved in the projects in various capacities. Collectively, the PAG represented a range of knowledge and skills sets related to MNP interventions. All 10 of the individuals we identified agreed to participate in either one of two ways.

1. Seven experts assisted by conducting mock interviews with our draft interview guide. They were assigned to assume the roles of different types of respondents (i.e., continuing user, noncontinuing user, or nonredeemer).
2. Three experts with research experience were asked to complete an in‐depth review of the interview guide, which involved reviewing each question for all types of respondents.

**Table 1:** Profiles of Project Advisory Group (PAG)

PAG member	Profile
1.	Licentiate degree in nutrition, MNP and IYC nutrition programme design, implementation and evaluation experience, knowledge of local context
2.	Master's degree in communication and development, experience in conducting formative research for the design and improvement of MNP and IYC nutrition programmes
3.	Doctoral degree in nutrition, experience in conducting focused ethnographic studies and quantitative surveys, knowledge of local context
4.	Doctoral degree in public health, experience in conducting studies using qualitative and quantitative methods, MNP and IYC nutrition programme design, implementation and evaluation experience
5.	Bachelor's degree in nutrition, IYC nutrition policy design and programme implementation experience, knowledge of local context
6.	Profession bachelor's degree in global nutrition and health, IYC nutrition programme implementation and evaluation experience
7.	Master's degree in management of international organizations, IYC nutrition programme implementation experience
8.	Master's degree in communication, public affairs and international relations, specialist in communication and project management
9.	Master's degree in social sciences, specialist in project management
10.	Master's degree in development studies, IYC nutrition programme implementation experience

*Note*. IYC: infant and young child; MNP: micronutrient powders.

To guide their feedback, we asked experts to pay particular attention to gaps in content, the flow and organization of questions, whether the questions were easily understood, as well as their overall impressions. The duration of mock interviews ranged from 30 to 60 min. Following the mock interviews, we also spent time debriefing with the PAG members, which was another aspect of our methodological innovation. Our discussions with the experts who served as in‐depth reviewers ranged from 90 to 150 min. The core team took handwritten notes based on their observations during the mock interviews, debriefing and in‐depth review discussions. The notes were then typed and shared with the experts to confirm that their comments were accurately recorded. Thematic analysis of the interview notes was conducted using a standard approach for analysing qualitative data. This analysis enabled us to identify main themes that emerged from the interviews with the members of the PAG (Miles, Huberman, & Saldaña, 2014). We completed all interviews with PAG members within 1 week.

### 2.5 Phase III: Core team revisions to the caregiver interview guide

Revising the caregiver interview guide was an iterative process based on expert feedback and core team reflection and discussion. All feedback and revisions were viewed in relation to the criterion that revisions would enhance our ability to collect essential information for the programmes' formative evaluation goal, while sustaining the principles of ethnography, with its emphasis on obtaining emic views and avoiding the imposition of investigators' and public health professionals' perspectives.

In Phase III of the interview guide development, the core team interacted with individual PAG members concerning potential modifications and re‐examined individual questions from the perspective of data management and analysis. The process of revising the interview guide (i.e., Phase III) took approximately 3 weeks to complete.

Finally, it is important to note that the development and critical review of the caregiver interview guide was undertaken in conjunction with the development of a general programme impact pathway model for caregiver MNP adherence as previously described by Tumilowicz et al. (2017).

## 3 RESULTS

Table 2 summarizes the results of the interview guide development process under four headings.

**Table 2:** Summary of results areas of the interview guide development process

Results area	Description
1. Gaps in content	Topical areas relevant to the MNP home fortification process evaluation found to be missing from the caregiver interview guide during pretesting.
2. Cultural adaptations and comprehension	Words, phrases, concepts, and questions that should be modified or eliminated for reasons related to translation or cultural appropriateness or to enhance the caregiver's understanding of the question's purpose/intent.
3. Interview guide structure and format	Alterations in question sequence to reduce potential for bias or enhance logical flow, recommendations for management of skip patterns, modifications in interview guide format to enhance usability by interviewer.
4. Differences in methodological approaches to the structure and content of questions	Findings related to the use of FES for formative evaluation purposes, including the scope and tone of interview guide questions.

*Note*. FES: focused ethnographic study; IYC: infant and young child; MNP: micronutrient powder.

### 3.1 Gaps in content

Phase II revealed several topics that needed to be explored more thoroughly than we had initially realized when we constructed the guide. These topics concerned the caregiver's experiences with intervention delivery, their sources of information about MNP and its recommended use, and the potential impact of social support networks on the caregiver's acceptance and utilization of MNP. For the Mozambique programme, which used a voucher system, a brief introductory module served to screen for MNP voucher receipt and redemption. However, many of the advisory team members felt that questions should be added to discover what the caregiver's experience was when she received and redeemed the voucher. Several PAG members noted a gap in investigating caregivers' exposure to various sources of information about MNP and how to use it. They recommended asking questions specifically about who explained to caregivers how to use MNP, what they were told, the setting in which they were told (i.e., individual conversation vs. group education), and what programme materials, if any, caregivers had received to aid in their understanding of the product. One expert with experience in maternal, infant, and young child nutrition programme development and implementation stressed the role social support can play in a caregiver's involvement with an intervention and recommended adding several questions to further explore the attitudes and behaviours of a caregiver's social support network to determine how those may have influenced her acceptance and utilization of MNP, either positively or negatively.

### 3.2 Cultural adaptations and comprehension

Pretesting in Phase II revealed words, phrases, concepts, and questions that needed to be modified. For instance, a note for the interviewers regarding the question, “Is there anything parents can give to help children grow?”, suggested that they might ask about “teas or tonics,” as an example. However, one expert with knowledge of the local culture noted that “tonics” would not translate well into the local language, nor would caregivers be likely to have the same understanding of “tonics” as the interview guide developers or perhaps any understanding of them at all.

### 3.3 Interview guide structure and format

PAG members provided insights into how the sequence of questions could be changed to (a) avoid the potential for bias; (b) enhance the logical flow of questions; and (c) minimize the frequency of skip patterns. They highlighted questions that might be redundant and could therefore frustrate participants. They suggested ways the interview guide format could be modified to improve the interviewers' experience. To minimize bias, PAG members suggested that follow‐up questions about respondents' sources of information about MNP should be asked *after* respondents were asked to describe behaviours with MNP, so that the line of questioning about sources of information and instructions did not influence how respondents described their own behaviours.

Although the core team recognized the complexity of the interview guide during the initial development phase and attempted to address this through format changes, our experts still raised concerns about frequent skip patterns. As the status of the respondent relative to use (continuing user, noncontinuing user, or nonredeemer) was not known before the interview began but emerged in response to the use questions, the original plan was to use a single caregiver interview guide with all the respondents. One PAG member recommended creating separate “passages” for different types of respondents in the module assessing the caregiver's behaviours with using MNP. These separate passages would eliminate the need for skips in the section on use behaviour, prevent confusion, and ease the burden on interviewers. We subsequently took the recommendation further and created separate interview guides for the different types of respondents. As soon as it was clear what type of user the respondent was, the interviewer simply reached for a separate interview guide.

However, the use of different guides did not entirely eliminate skip patterns because even caregivers in the same intervention respondent type category had unique experiences and perspectives to share that needed to be embraced, anticipated, and accounted for.

One PAG member, who conducted an in‐depth review of the interview guide, recommended placing all questions regarding intervention delivery in Mozambique, including voucher receipt and redemption in the same module to make it easier for caregivers, rather than asking intervention delivery questions in both the opening and closing modules, as was the initial plan.

Related to the interview guide structure and format, and similar to the core team's own discussions, experts shared their ideas about how to handle “yes/no” questions. We initially included “yes/no” questions and skip patterns to distinguish who needed to answer which questions. However, expert feedback about the complexity of skip patterns and discussions about how to best elicit all the information for the formative evaluation led us to rethink these questions and the interviewer instructions that came with them. We recognized that not only would additional skip patterns be complicated for the interviewers but they could also prevent the interviewer from collecting valuable information. Thus, instead of using “yes” and “no” options on the interview guide, with skip patterns and separate follow‐up questions (which is more aligned with quantitative survey formats), we decided that “yes” and “no” answers should be avoided in favour of questions that provided insights about the caregiver's experiences. For example, instead of prefacing with, “Do you remember the first time your child got food with MNP?” the question directly asks about the experience, “Thinking back to the first time your child got some food with MNP, do you remember how he or she took it; what the child's response was to the food?”

Members of the PAG raised concerns about whether the interviewers would be able to elicit the necessary information. They also emphasized the need for interviewers to have a solid understanding of the technical aspects of MNP and the intervention, as well as strong interviewing skills to effectively probe and prompt on answers to open‐ended questions.

Finally, with respect to format, another finding of note is that none of the experts expressed concern about the use of questions that were framed in a “survey‐like format” in what was positioned as “a qualitative study.”

### 3.4 Differences in methodological approaches to the structure and content of questions

A primary theme in the feedback from the expert team was criticism of the “conversational tone” of the open‐ended questions. A second, related concern was the balance between “broadness” and “specificity” of questions. The differences in methodological approaches were revealed in several ways, outlined below.

A central piece of feedback from one expert with extensive MNP programming and research experience was to make the interview guide more straightforward by simplifying and condensing questions and removing extraneous instructions because introductory language or “pleasantries” ran the risk of “getting lost in translation.” For example, instead of phrasing a question as: “You have told me what happens to leftover food with MNP, but I also wonder, does anyone else in the family besides (child name) also get (MNP product name)?” a PAG member suggested we simply ask: “Does anyone else in the family get (MNP product name)?”

Following basic ethnographic techniques, the core team explicitly crafted the questions to elicit “emic” perspectives and reduce the influence of the investigators' or interviewers' perspectives. However, PAG experts made numerous suggestions to rephrase questions to be more direct, which in some instances led to tension between maintaining an ethnographic approach but ensuring specific information of interest was still elicited. For example, as part of exploring caregivers' interpretations and perceptions of MNP, we asked: “Are there times when it is particularly more important to give infants and young children vitamins?” One expert team member suggested asking: “Did you give your child MNP the last time he or she was sick?”

There were also tensions regarding the instructions for interviewers on using probes. Relating to the concern about information to improve the programmes, there were lively discussions about whether that information would come out naturally as part of a caregiver's answer to a broader question followed by probing, or whether separate, more specific questions were required to elicit the desired information. Some PAG members were concerned about relying too much on probes and follow‐up questions for fear that the suggested probes would be overlooked or forgotten by interviewers in the “heat of the interview.”

Discussions between PAG members and core team members also concerned the issue of appropriate specificity of the probes themselves. Some thought it was important for probes to be explicit so that interviewers would know the exact information that was important to obtain, whereas others promoted the idea that the listing of potential probes should be more general to avoid unduly influencing respondents.

An example that illustrates both “specificity” and “probe issues” in experts' feedback is a question asking caregivers to describe how they prepared foods with MNP. We originally framed the question as follows:

Please tell me the steps you followed when you prepared the food with MNP. I'd like to understand a little more about how you mixed the MNP with (food name or foods) when you fed it to (child name). Can you explain to me how you did this?

We wrote instructions to help interviewers lead the respondents through the step‐by‐step process, such as asking “and then what?” after the caregiver described each step. We listed topical probes to capture different parts of the preparation process, such as the consistency and portion of food to which MNP was added, if the whole MNP sachet was used, and at what point in the food's preparation the MNP was added. The PAG members were not uniform in their suggestions concerning this important question. The suggestions included (a) making each of the preparation step probes into separate questions, (b) making the questions more specific to ensure the information was obtained, and (c) providing probing suggestions that consisted of few words to cue interviewers to topics of interest or, alternatively, writing out the probes as full questions so interviewers knew exactly what to say. For example, one PAG member suggested we ask: “Did you add the MNP to the food while it was still cooking?” In the final version, we included the probe: “Do you remember at what point in the food preparation or cooking process the MNP was mixed in with the food?” We reasoned that we would still learn when she added the MNP, but the information could be elicited in a less biased and more emic way. In the analysis, the information obtained from this question turned out to be essential to determine whether caregivers actually followed the preparation instructions they should have been given by the health workers.

In contrast to the foregoing concerns, one PAG member with extensive MNP research experience, and who was also more familiar with a focused ethnography approach and the focused ethnographic study on infant and young child feeding manual (G. H. Pelto & Armar‐Klemesu, 2014), provided suggestions about how the interview guide could be made even more ethnographic. For example, in the module focused on the caregivers' experiences with MNP, the expert suggested adding, “What was your experience like when you first started using (MNP product name)?” The purpose of this question was to provide the respondent with an opportunity to express her unique experience.

## 4 DISCUSSION

The summary of results in Table 2 shows that the addition of Phase II (the PAG and its specific activities) played a vital role in shaping the development of the interview guide. The PAG, with its diversity of expertise and experience, provided an important complement to the experience of the core team. In fact, it proved so valuable for development of the tool (both content and logistics) that we regard it as a significant finding with respect to the practice of implementation research in nutrition. The value of advisory groups in other types of nutrition research has long been recognized, and we suggest that it is time to incorporate it into implementation research. Thus, based on our experiences in our MNP formative evaluation study, we encourage nutrition implementation researchers to consider including a PAG and a Phase II component in their research design, to the extent that this is logistically, organizationally, and fiscally possible. Based on our experience, we recommend a PAG, which incorporated experts with a depth of knowledge about the intervention, research methodologies, and local context.

The differentiation within the sample of caregivers is another important finding that needs to be highlighted. This differentiation is intrinsic to an intervention that involves interactions between programmes and intended beneficiaries. At the beginning of an intervention, “the beneficiaries”—in this case the children who could benefit from MNP and their caregivers, who are responsible for the steps in the household delivery system from acquiring the MNP to preparation and feeding—tend to be viewed as a uniform group. But as the intervention progresses, they become differentiated in relation to their responses. For both the Ethiopia and Mozambique intervention studies, we labelled the differentiation of the groups as continuing users, noncontinuing users, or nonredeemers.

In Phase I, the initial planning of the formative evaluation, the core team did not recognize the full logistic and training implications of differentiation, particularly in relation to issues of interview guide format (skip patterns) and question construction (probing). In Phase II, with the essential inputs from the PAG, the implications of differentiation became fully apparent. After the fact, it seems obvious, but the implications of differentiation, particularly the methodological implications, only emerged over the course of developing the data collection instrument. In Phase III, we collectively developed solutions to the challenges of differentiation with respect to the structure of the interview schedules, as well as interviewer recruitment, training and monitoring, and resource allocation.

The study results illustrate the ways in which some of the fundamental features of ethnography are different from survey‐type research. The discovery that some PAG members were uneasy with, or even negative about, some of the features of good qualitative interview techniques was surprising. It is an important finding and raises larger issues that require exploration to identify the reasons for the tensions related to methodological approaches and what can be done to ameliorate them.

Throughout the process of developing and implementing the research, the ethnographically trained and oriented members of the core team were committed to maintaining the principles of ethnographic interviewing to accurately capture caregivers' experiences with MNP. When we changed questions and/or probes, the end results were closer to the ethnographic rather than the quantitative survey end of the spectrum. The interview guides developed for Mozambique through the process described here is available in Supporting Information.

Beyond the basic premise that caregivers are often best placed to offer insights into whether or how a nutrition intervention and its mode of delivery are functioning, there are inherent advantages of using of ethnographic methods in formative evaluation (Fetterman, 1984; Patton, 2002). Among the benefits of ethnographic interviewing techniques are technical issues of data validity (e.g., avoiding the consequences of assuming incorrectly that the interviewee interprets the questions in the way you intend, or the likelihood you will receive answers the interviewees think you are looking for, or the likelihood that interviewees will give you what they have heard from their frontline worker contact; S. L. Schensul et al., 1999). These techniques also reduce the danger that the phrasing of your questions impose biomedical nutrition constructs on the respondents that do not reflect their cultural constructs and cognitive organization, and thereby reducing the opportunity to gain insights that are important for understanding their reactions and experiences and taking corrective actions based on the formative evaluation results. In addition to technical and scientific issues, another reason to preserve a conversational interaction is to assist interviewers to establish and maintain rapport with respondents (Spradley, 1979). This is not only important in terms of the role of rapport in yielding richer insights, it is also ethically important to create a positive experience for study participants.

As noted above, the research results we obtained by using an ethnographic approach are described in other papers in this supplement (Pelto et al., 2019; Tumilowicz, Habicht, et al., 2019; Tumilowicz, Vossenaar, et al., 2019). We gained insights from the perspective of caregivers that have not previously been reported in the literature. For example, in Ethiopia, we learned that caregivers face new sets of challenges to continue feeding MNP through their children's second year of life as they experience periods of illness and poor appetite, and progress through developmental stages that affect feeding behaviours (G. Pelto et al., 2019). In Mozambique, we identified challenges in access to obtaining the supplement that had not been foreseen in the design and implementation of the delivery system (Tumilowicz, Vossenaar, et al., 2019).

There is a relatively large body of literature of valuable ex post facto analyses of how well‐intentioned nutrition and public health interventions failed to achieve their intended impact because of cultural, social, and behavioural factors in both the delivery systems and utilization systems in which the interventions took place. This scientific literature has a long‐time depth, dating back many decades, and continues to grow as social science investigators conduct evaluations of nutrition and public health interventions. However, at present, there are few published examples of formative evaluations that systematically explore caregivers' perspectives to understand how delivery and utilization processes affect programme outcomes or impacts. This lacuna is a significant gap in supporting the application of ethnography in implementation research.

In our view, ethnographically trained implementation scientists need to do a better job of documenting the benefits of expanding and employing their research modalities, explaining the different types of techniques of their mixed method approach, and addressing the concerns of quantitative researchers. At the same time, quantitatively oriented investigators need to be better informed about the benefits and contributions of ethnography. There are, undoubtedly, multiple reasons for the slow progress on application of mixed methods. Some of these involve fundamental beliefs about what constitutes science and scientific rigour. Some of it can be attributed to the “silos” that are created by social, academic, and institutional structures, as well as communication modalities and channels.

A systematic and empirical exploration of the reasons for the study findings concerning the tensions that were revealed in Phase II of the study is beyond the scope of this paper. However, we hope that these results, which highlight the need for better communication among implementation scientists working in the field of nutrition interventions, will lead to further discussions and new actions to support sound and effective formative evaluation, as well as other aspects of implementation research in nutrition.

## CONFLICTS OF INTEREST

The authors report no conflicts of interest.

## CONTRIBUTIONS

CHS, AT, and GHP developed the paper concept; CHS conducted the PAG pretesting and analysis and wrote the first draft of the manuscript; AT and GHP provided intellectual content; all authors read and approved the final version submitted for publication.

## Supporting information

Data S1: Supporting information

Data S2: Supporting information

Data S3: Supporting information

Data S4: Supporting information
