# Supplementary material for: Designing an ethnographic interview for evaluation of micronutrient powder trial: Challenges and opportunities for implementation science
Source: Matern Child Nutr. 2019 Oct 17;15(Suppl 5):e12804. doi: 10.1111/mcn.12804 (PMC6856841; doi:10.1111/mcn.12804)
Supplement: Supplementary file 2 — Data S2: Supporting information [file MCN-15-e12804-s002.docx]

**Focused Ethnographic Study Protocol for the Mozambique Process Evaluation in Mozambique – DEFAULTER PROTOCOL**

**The questions in Modules 3D and 4D are specifically for caregivers who redeemed the voucher and tried Vitamais for a period of time but are currently no longer using it. Defaulters who never tried giving Vitamais but still redeemed the voucher will start at Module 5D.**

| **Module 3D: Caregiver Behaviours with Vitamais** | | | |
| --- | --- | --- | --- |
| **MODULE INTRODUCTION: “**Thank you for telling me about your experience with the voucher, getting Vitamais at the store and more about why you are no longer giving Vitamais to (child name). I’d like to ask you some questions now about what happened when you used to give Vitamais.” | | | |
|  | **QUESTION** | **ANSWER** | **SKIP** |
| Q301. | Are you currently breastfeeding? | 00 = No | |
|  |  | 01 = Yes | |
| Q302. | I know it might be hard to remember exactly what he/she ate, but can you tell me what you gave (child name) to eat and drink yesterday, from the time he/she woke up until he/she went to sleep for the night?  *Probe to make sure you get a list of everything the child consumed throughout the day and evening.* |  | |
| Q303. | When you gave Vitamais to your child, how many times did you try it? How many times did you give Vitamais to him/her?  *Probe for how many times per week to help quantify.*  *Probe to see if she used to give it every day. If she didn’t give it every day, probe to find out the reasons why.*  *If she says she forgot to give it, probe to find out what helped her remember to give it.* |  | |
| Q304. | Do you remember what foods you put it into?  *Probe for as many foods as she mixed Vitamais into that she can list.* |  | |
| Q305. | I’d like to understand a little more about how you mixed the Vitamais with *(food name or foods)* when you fed it to (child name). Can you explain to me how you did this?  *Allow caregiver to explain. You can use “and then what?” probes to help her tell the step-by –step process she followed. You might have to ask the following questions if you don’t have answers to any of the following:*   1. *Could you tell me about the consistency of the food the Vitamais was mixed into? Was it thick or thin? Did it fall off of the spoon/slide through your fingers easily?* 2. *Do you remember at what point in the food preparation or cooking process you mixed the Vitamais into the food?* 3. *How much of the Vitamais packet did you mix into the food?* 4. *Could you tell me about how much food the Vitamais was mixed into*? |  | |
|  |  | 01 = Put the FULL packet into the FULL PORTION portion I expected child to eat | **🡪Q307** |
|  |  | 02 = Put the FULL packet into a SMALLER AMOUNT of food than I expected child to eat | **🡪Q307** |
|  |  | 03 = Put PART of the packet into the FULL PORTION I expected child to eat |  |
|  |  | 04 = Put PART of the packet into SMALLER AMOUNT of food than I expected child to eat |  |
| Q306. | Can you tell me what happened to the powder that you didn’t mix into the food?  *Probe to see if the food was*  *-saved and given to the child later that day (and if so, with what food was it mixed and how)*  *- saved to give to the child today*  *-fed to someone else (and who)*  *- fed to animals*  *-thrown away.*  ***Be sure you know what happened to the food with Vitamais in it.*** |  | |
| Q307. | Did you prepare the food any differently because Vitamais was being mixed in than you would have prepared it otherwise?  *If yes, probe for what the differences in preparation were.* |  | |
| Q308. | Do you know how much time passed between mixing the food with the Vitamais and (child name) eating it?  *If yes, probe for how much time passed.* |  | |
| Q309. | When you think about food that you put the Vitamais into, how many times would you say that (child name) **didn’t** eat all of it? |  | |
| Q310. | Why do you think (child name) sometimes didn’t eat all of the food?  *Probe for if not eating all of food is normal for this child.* |  | |
| Q311. | What would happen to the food that had the powder mixed in that (child name) didn’t eat?  *Probe to see if the food was saved and given to child later, if it was fed to someone else (and who), if it was fed to animals or thrown away.*  ***Be sure you know what happened to the food with Vitamais in it.*** |  | |
| Q312. | Thinking about how you fed (child name) Vitamais, are there any other foods you used to put Vitamais into?  *If yes, probe for what these foods are.* |  | |
| Q313. | What are the reasons you mixed it with those foods?  *Probe for as many reasons as she can provide.* |  | |
| Q314. | Could you tell me about how you learned to use Vitamais for feeding to your child?  *Probe for if she learned from certain people, Vitamais packaging or any educational materials.*  *If she lists multiple sources of information, probe for which one she thought was the most helpful.* |  | |
| Q315. | You’ve told me what happened to leftover food with Vitamais, but I also wonder, did anyone else in the family besides (child name) also eat Vitamais?  *If yes, probe for why she gave Vitamais to these other family members? How often did that happen?* |  | |
| Q316. | Thank you for explaining all about what you did when you fed (child name) Vitamais. Did anyone else in the household also give (child name) Vitamais? When you were away and (child name) was being taken care of by someone else, did they ever give him/her Vitamais?  *Probe for who else fed the child Vitamais, how often, who provided instructions to this person for how to use Vitamais.* |  | |

| **Module 4D: Caregiver Experiences Feeding Vitamais** | | | |
| --- | --- | --- | --- |
| **Note:** *This module shifts the emphasis from the caregiver’s behaviours in feeding Vitamais to her child to her experiences. Of course the behaviours you have just discussed will also be influenced by her past and present experiences, so she may have already told you quite a bit. In this case, you need to choose the questions from this module carefully so she doesn’t have to say, “I’ve already told you that.”*  *It is a shorter module, and the purposes of the questions in it are to get a better picture of what the respondent’s experiences have been. The answers to her questions will be heavily influenced by the age of her child and how long she has been using Vitamais or how long ago she decided not to use it.*  **MODULE INTRODUCTION: “**Now I’d like to ask you some questions about your experiences feeding (child name) foods with Vitamais mixed in. Again, there are no right or wrong answers. I’m really interested in hearing about your own personal experiences.” | | | |
|  | **QUESTION** | **ANSWER** | **SKIP** |
| Q401. | Do you sometimes taste (child name)’s food before you give it to him/her? | 00 = No | |
|  |  | 01 = Yes | |
| Q402. | Thinking back to when you started putting some Vitamais into (child name)’s food, did you taste it before you gave it to (child name)?  *If yes, probe to see if she thought there was anything different about the taste and whether or not it was a good, bad or neutral difference.*  *Ask these questions if caregiver hasn’t mentioned anything about* ***smell****,* ***look*** *or* ***texture****.*   1. *Did the food smell differently than normal?* 2. *Did the food have a different look to it than normal?* 3. *Did the food’s texture change after mixing in the Vitamais?*   *Make a note if she says any of these differences changed her perception of Vitamais positively or negatively.* |  | |
| Q403. | Thinking back to when (child name) first started eating food with Vitamais, do you remember how he/she reacted?  *Probe for whether it was the same as usual or different than to how he/she took foods before Vitamais.*  *If it was different, probe for what the child’s response was.*  *Probe for if child’s response to foods with MNP changed over time.* |  | **If child’s response was the SAME as usual or was POSITIVE**  **🡪 Q405**  **If child’s response was NEGATIVE 🡪 Q404** |
| **Important Note: If the caregiver asks why you are asking these questions, you need to have a simple explanation. For example, you could say, “Well some people have said they can taste it, but many people say no, so I just wanted to find out about your experience.”** | | | |
| Q404. | Do you remember what *you* did when (child name) responded to the food that way?…[use mothers words]  *Probe to find out if the caregiver’s action was successful* ***from her perspective*** *and whether she continued with that action.*  *If her action was to stop giving it and she expresses the idea that this was fine with her, this is important to note.* |  | |
| Q405. | Did you notice any changes in (child name) when you were feeding him/her foods with Vitamais?  *If yes, ask caregiver to describe changes.* |  | |
| Q406. | Is there anything else you’d like to share about your overall experience using Vitamais that we haven’t talked about? |  | |

| **Module 5D: Caregiver Health Beliefs and Knowledge** | | | |
| --- | --- | --- | --- |
| **Note: Defaulters who never tried giving Vitamais but still redeemed the voucher will start here.**  **MODULE INTRODUCTION: “**Now I’d like to get your opinions about whether there are things parents can do to help children grow well and keep them from getting sick. I’m really interested in your opinion. There are not right or wrong answers because every mother and father has their own views about this and their own experience.**”** | | | |
|  | **QUESTION** | **ANSWER** | **SKIP** |
| Q501. | I’ll start by asking you whether there *is* anything mothers and fathers can do for their child’s health or is it mostly outside of their control? | 01 = No, it is mostly out of parents’ control |  |
|  |  | 02 = Yes, there are things parents can do to keep their child healthy | **🡪 Q505** |
| Q502. | Could you tell me more about why is there nothing parents can do to help their child’s health? |  | |
| Q503. | So we were just talking about keeping children **healthy**, but I’m wondering if there is anything you can ***give*** to help your child ***grow***?  *Can say “anything the child could eat, drink, swallow or receive into their body,” if caregiver needs an example.*  *Probe for why these things are good for child growth.*  *Make a note about whether she specifically mentions “vitamins,” or whether she says something else to refer to Vitamais’ contents, such as “things it contains,” “medicines,” “ingredients,” etc. or other components.* |  | **If caregiver EVER gave Vitamais  🡪 continue at Q504**  **If caregiver NEVER gave Vitamais  🡪 Module 6D Caregiver Reflections on Vitamais** |
| Q504. | You said earlier that you used to feed Vitamais to (child name). Can you tell me why you fed it to him/her? Why did you decide to try Vitamais? |  | **🡪 Module 6D Caregiver Reflections on Vitamais** |
| Q505. | So what are the kinds of things that mothers and fathers can do to improve their child’s health?  *If caregiver says something about “giving good food” or “proper feeding,” probe for what that means.* |  | |
| Q506. | What are the reasons these things help improve a child’s health? |  | |
| Q507. | So we were just talking about keeping children **healthy**, but I’m wondering if there is anything you can ***give*** to help your child ***grow***?  *Can say “anything the child could eat, drink, swallow or receive into their body,” if caregiver needs an example.*  *Probe for why these things are good for child growth.*  *Make a note about whether she specifically mentions “vitamins,” or whether she says something else to refer to Vitamais’ contents, such as “things it contains,” “medicines,” “ingredients,” etc. or other components.* |  | **If caregiver EVER GAVE Vitamais  🡪 continue at Q508**  **If caregiver NEVER GAVE Vitamais**  **🡪 Module 6D Caregiver Reflections on Vitamais** |
| Q508. | What about Vitamais? Can you tell me why you gave it to him/her? I know you’re not using it anymore, but why did you consider giving Vitamais a try? |  | |

| **Module 6D: Caregiver Reflections on Vitamais** | | |
| --- | --- | --- |
| **MODULE INTRODUCTION: “**You have provided so much helpful information so far, and I really appreciate that. If it’s okay, I have just a few more questions I would like to get your views on before we end with some questions about your household.” | | |
| Q601. | As you know, there are vouchers for Vitamais only for children who are between 6 and 23 months. I’m wondering if you think it is a problem that there are no vouchers for children once they are two years old? Or is that okay?  *Probe for reasons why caregiver thinks it is or isn’t a problem that Vitamais is only for children 6-23 months old.* |  |
| Q602. | What do others in your household and community think about Vitamais?  *Probe for as many opinions about Vitamais as caregiver can provide.*  *Probe for the reasons for those opinions and if the opinions have changed over time.* |  |
| Q603. | Based on your experience, would you recommend Vitamais to other mothers and caregivers for their children? | 00 = No |
|  |  | 01 = Yes |
| Q604. | Is there anything that might be done to make Vitamais better or something families would like to use?  *Probe for what the caregiver thinks those things are.* |  |
| Q605. | Do you have any final thoughts you would like to share with us that we haven’t already talked about? |  |

| Module 7D. Socio-demographic Information | | | | |
| --- | --- | --- | --- | --- |
| **MODULE INTRODUCTION:** “Thank you so much for all of the thoughtful information you’ve given me today. It makes a big difference for all of us to understand, first-hand, what mothers’ experiences with the vouchers and Vitamais are, how they have solved problems and how they’ve decided to give it to their babies or decided that it is better not to give it to them. I know this will be very helpful. I just have a few final questions about your household.” | | | | |
|  | **QUESTION** | **ANSWER** | **CATEGORIES AND CODES** | **SKIP** |

| Q701 | How many people, in total, currently live in your household?  HOUSEHOLD IS DEFINED AS "ALL PEOPLE LIVING IN THE HOUSE FOR THE LAST 6 MONTHS AND ATE ON THE SAME POT " | TOTAL |  |
| --- | --- | --- | --- |
|  |  | REFUSES TO ANSWER | 97 |
|  |  | DOES NOT KNOW | 98 |
| Q702 | how many children under 5 years old live in the household?  including the target child | TOTAL NUMBER OF CHILDREN |  |
| Q703 | Of the children living in the household, who is in the age range between 6 to 23 months?  INCLUDING THE TARGETED CHILD, IF APPLICABLE | TOTAL NUMBER OF CHILDREN  IF NONE, '00' |  |
| Q704 | Of the children living in the household, who is in the age range between 24 to 59 months (2 to 5 years old)?  INCLUDING THE TARGETED CHILD, IF APPLICABLE | TOTAL NUMBER OF CHILDREN  IF NONE, '00' |  |
| Q705 | What is the native language of the head of the household? | SENA | 01 |
|  |  | NDAU | 02 |
|  |  | SHONA | 03 |
|  |  | PORTUGUESE | 04 |
|  |  | OTHER (SPECIFY): _________________________ | 96 |
| Q706 | What is your relation to (CHILD NAME)? | MOTHER | 01 |
|  |  | GRANDMOTHER | 02 |
|  |  | BROTHER | 03 |
|  |  | OTHER (SPECIFY): _________________________ | 96 |
| Q707 | How old are you?  INDICATE THE AGE OF THE INTERVIEWEE | AGE |  |
|  |  | REFUSES TO ANSWER | 97 |
|  |  | DOES NOT KNOW | 98 |
| Q708 | Finally, I’d like to know about your schooling. Did you go to school? Please tell me what schooling you have had. | ADULT LITERACY PROGRAM | 01 |
|  |  | PRIMARY EP1 (GRADE 1 TO 5) | 02 |
|  |  | PRIMARY EP2 (GRADE 6 TO 7) | 03 |
|  |  | SECONDARY ESG1 (GRADE 8 TO 10) | 04 |
|  |  | SECONDARY ESG2 (GRADE 11 TO 12) | 05 |
|  |  | ELEMENTARY TECHNICAL EDUCATION | 06 |
|  |  | BASIC TECHNICAL EDUCATION | 07 |
|  |  | MIDDLE TECHNICAL EDUCATION | 08 |
|  |  | TEACHING COURSE | 09 |
|  |  | SUPERIOR | 10 |
|  |  | OTHER (SPECIFY): _______________________ | 96 |
|  |  | DOES NOT KNOW | 98 |
|  |  | REFUSES TO ANSWER | 97 |
|  |  | DOES NOT KNOW | 98 |

**“This is now the end of the interview. Thank you again so much for your participation and your time. It is so helpful for us to get your perspective and learn more about your experiences as well as those of other mothers, grandmothers and caregivers in the community that we have had or will have the opportunity to talk with. All of the information you have shared today will help us make programs better for you, your children, your family and your community. Thank you!”**
