# Supplementary material for: Designing an ethnographic interview for evaluation of micronutrient powder trial: Challenges and opportunities for implementation science
Source: Matern Child Nutr. 2019 Oct 17;15(Suppl 5):e12804. doi: 10.1111/mcn.12804 (PMC6856841; doi:10.1111/mcn.12804)
Supplement: Supplementary file 3 — Data S3: Supporting information [file MCN-15-e12804-s003.docx]

**Caregiver Interview Guide for the GAIN Mozambique Process Evaluation**

*Good morning/afternoon, my name is ________ and I am a member of the GAIN and COWI Mozambique team that is conducting a study about young child feeding and nutrition in Mozambique. GAIN is an international organization working to achieve a world without malnutrition. COWI is a Mozambican consultancy company, hired by GAIN to conduct this study. As part of this study, we would like to talk to people in the community who have received these vouchers (show example of paper and electronic voucher). We learned from the program that your household may have received one.*

*[Confirm with caregiver that she received a voucher. If she did not, respectfully end the interview.
If she did receive the voucher, continue on.]*

*The interview will last about 1 hour. Your participation is voluntary and if by any chance you are not comfortable to answer a question, please feel free to say and we will proceed to the following question. Everything in this conversation is confidential and will not be shared with people outside our study team. Can we proceed?*

**ask to speak with the Person mainly responsible for feeding the selected child of 6 to 23 Months.**

**in the case of granted consent, proceed with the interview.**

**IF CONSENT FOR INTERVIEW IS NOT GIVEN, STOP THE INTERVIEW.**

| Module 0: Identification Information | | | |
| --- | --- | --- | --- |
| Q001. | Consent to conduct interview*: YES / NO | Q008. | District: |
| Q002. | Child Number:  I___I___I District  I___I___I Health area  I___I___I Village  I___I___I Household | Q009. | Health Area: |
| Q003. | Child’s Name: | Q010. | Village: |
| Q004. | Age range **1 =** (6-11 mo) or **2 =** (12-23 mo ): **1 2** | Q011. | Interviewer Code: |
| Q005. | Mother’s Name: | Q012. | Starting Time: |
| Q007. | Date of Interview: __ __ / __ __ / __ __ __ __ (DD/MM/YYY) | Q013. | End Time: |

| Module 1. Child Identification Information | | | | |
| --- | --- | --- | --- | --- |
| **NOTE:** *You do not need to confirm the birth date with a document unless the caregiver is unclear or unsure about the child’s birth date. Her report is sufficient because anthropometry is not involved with this interview.*  **INTRODUCTION: “**I’d like to start by asking a few questions about your youngest child’s age.**”** | | | | |
|  | **QUESTION** | **ANSWER** | **CATEGORIES AND CODES** | **SKIP** |

| Q101 | Date [CHILD’S NAME] was born (day, month and year?  If the mother gives the birth month and year immediately and does not hesitate, you do not need to confirm Against any other documentation  If the mother hesitates to recall the month and/or year, confirm birth date with one of the following:  -Birth certificate  -Health card  -any other written source caregiver may have  -use a CALENDAR OF EVENTS TO ESTIMATE THE MOST CORRECT DATA REGARDING BIRTH/AGE. | DAY |  | |
| --- | --- | --- | --- | --- |
|  |  | MONTH |  | |
|  |  | YEAR |  | |
|  |  | DOES NOT KNOW | 98 | |
|  |  | IF THE INTERVIEWEE DOES NOT KNOW THE COMPLETE DATE (E.G. REMEBERS ONLY THE MONTH AND YEAR) OR IF THE DATE IS INCOMPLETE ON THE DOCUMENT, WRITE:  99 (LACKING DAY OR MONTH)  OR 9999 (LACKS YEAR) | | |
| Q102 | INDICATE HOW YOU HAVE CONFIRMED THE INFORMATION REGARDING THE BIRTH DATE | CAREGIVER’S REPORT | 01 | |
|  |  | BIRTH CERTIFICATE | 02 | |
|  |  | HEALTH CARD | 03 | |
|  |  | CALENDAR OF EVENTS | 04 | |
|  |  | NOT CONFIRMED WITH ANOTHER SOURCE | 05 | |
|  |  | ANOTHER SOURCE (SPECIFY): __________________________ | 96 | |
| Q103 | BASED ON THE CHILD’S BIRTH DATE (**Q101**), DOCUMENT THE CHILD AGE RANGE | 6 TO 11 MONTHS | 01 |  |
|  |  | 12 TO 23 MONTHS | 02 |  |
|  |  | UNDER 6 MONTHS | 03 | **NOT ELIGIBLE CHILD.** STOP INTERVIEW |
|  |  | OVER 23 MONTHS | 04 | **NOT ELIGIBLE CHILD.** STOP INTERVIEW |

| Module 2: Program Delivery & Vitamais Utilization Screening | | | |
| --- | --- | --- | --- |
| **NOTE:** *You will need to use the “Caregiver Interview Tracking Form” with this module to make sure you are interviewing only the number of respondent types in each community needed.*  *If you have not previously interviewed a caregiver meeting the same criteria as the caregiver you are currently interviewing (e.g., an Adopter with child 6-11 months old in Beira high redemption rate health area and community #1 OR you still need to interview a second Adopter with a child 6-11 months old), continue the interview with the appropriate respondent type protocol. After the interview is complete, make a tally or write “1”/”2” in the appropriate box according to what type of respondent the caregiver was and in which community.*  *If the caregiver you are currently interviewing matches the same criteria as a caregiver you have already fully interviewed (e.g., another Defaulter in Beira high redemption rate health area and community #1), you DO NOT need to continue interviewing. Make a tally in the “Not needed because sample size reached” column and then respectfully end the current caregiver interview and thank them for their time and insights.*  **Introduction:** “We are very interested in hearing more about your experiences with the voucher you received and what happened after that.” | | | |
|  | **QUESTION** | **ANSWER** | **SKIP** |
| Q201. | Could you tell me about your experience in actually getting the voucher? What happened when you got the voucher?  *Probe for*  *-how she knew about or was approached with the voucher*  *-if she received any information (and by who) on what to do with the voucher*  *-if she knew what she was supposed to do with the voucher after she got it*  *-if the process was easy or difficult in any way* |  | |
| Q202. | I’m wondering if you took the voucher to a store (Troca Aki) to redeem it? | 00 = No |  |
|  |  | 01 = Yes | **🡪 Q205** |
| Q203. | Could you tell me some of the reasons you did not redeem the voucher?  *Probe for as many reasons as the caregiver can provide.* |  | |
| Q204. | Is there anything that might be done to make the voucher better or easier to redeem or use?  *Probe for what the caregiver thinks those things are.* |  | **Then 🡪 Non-Redeemer Protocol, Module 5NR** |
| Q205. | What was your experience like redeeming the voucher? What happened when you took your voucher to the store?  *Probe for*  *-what she received in exchange for the voucher and if it was what she expected*  *-whether she felt like she knew what she was supposed to do with the voucher*  *-if it was easy or difficult to redeem the voucher and why*  *-how much time passed between her getting the voucher and redeeming it* |  | |
| Q206. | What are the reasons you redeemed the voucher?  *Probe for as many reasons as she can provide.* |  | |
| Q207. | What could be done to make it easier for you to redeem the voucher? |  | |
| “Thank you for telling me about your experience with the voucher. Now I’d like to ask you a few questions about what happened after you redeemed the voucher and feeding (child name).” | | | |
| Q208. | I’d like to know what happened with the Vitamais you got at the store. Did you try giving it to (child name)? | 00 = No |  |
|  |  | 01 = Yes | **🡪Q210** |
| Q209. | Could you tell me more about why you did not feed (child name) Vitamais after you redeemed your voucher? |  | **🡪 Defaulter Protocol, Module 5D** |
| Q210. | Do you remember how you gave it to him/her the first time? | 00 = No | |
|  |  | 01 = Yes | |
| Q211. | Are you still giving it to him/her? | 00 = No |  |
|  |  | 01 = Yes | **🡪 Adopter Protocol, Module 3A** |
| Q212. | Could you tell me more about why you are not giving Vitamais?  *Probe for how long ago she stopped using it.*  ***It is important to determine whether she ran out of her supply or not.*** |  | **If caregiver says she RAN OUT of Vitamais**  **🡪 continue at Q213**  **If caregiver DIDN’T RUN OUT, stopped giving for another reason  🡪 Defaulter Protocol, Module 3D** |
| Q213. | I’m wondering why you ran out of Vitamais. Could you tell me more about that?  *Probe for*  *-how long ago she ran out*  *-WHEN, HOW and WHERE she can get another voucher for more Vitamais*  ***It is important to determine if***  ***-1 packet was given per day until supply ran out***  ***-more than 1 packet was given to the child per day***  ***-the MNP was shared with other  family members or friends***  ***-it was sold*** |  | **Then 🡪 Adopter Protocol, Module 3A, Q304** |
