# Supplementary material for: Designing an ethnographic interview for evaluation of micronutrient powder trial: Challenges and opportunities for implementation science
Source: Matern Child Nutr. 2019 Oct 17;15(Suppl 5):e12804. doi: 10.1111/mcn.12804 (PMC6856841; doi:10.1111/mcn.12804)
Supplement: Supplementary file 4 — Data S4: Supporting information [file MCN-15-e12804-s004.docx]

**Focused Ethnographic Study Protocol for the Mozambique Process Evaluation in Mozambique – NON-REDEEMER PROTOCOL**

**The questions in these modules are specifically for caregivers who did not redeem a voucher and thus did not use Vitamais.**

| **Module 5NR: Caregiver Health Beliefs and Knowledge** | | | |
| --- | --- | --- | --- |
| **MODULE INTRODUCTION: “**Now I’d like to get your opinions about whether there are things parents can do to help children grow well and keep them from getting sick. I’m really interested in your opinion. There are not right or wrong answers because every mother and father has their own views about this and their own experience.” | | | |
|  | **QUESTION** | **ANSWER** | **SKIP** |
| Q501. | I’ll start by asking you whether there *is* anything mothers and fathers can do for their child’s health or is it mostly outside of their control? | 01 = No, it is mostly out of parents’ control |  |
|  |  | 02 = Yes, there are things parents can do to keep their child healthy | **🡪 Q504** |
| Q502. | Could you tell me more about why is there nothing parents can do to help their child’s health? |  | |
| Q503. | So we were just talking about keeping children **healthy**, but I’m wondering if there is anything you can ***give*** to help your child ***grow***?  *Can say “anything the child could eat, drink, swallow or receive into their body,” if caregiver needs an example.*  *Probe for why these things are good for child growth.*  *Make a note about whether she specifically mentions “vitamins,” or whether she says something else to refer to Vitamais’ contents, such as “things it contains,” “medicines,” “ingredients,” etc. or other components.* |  | **🡪 Module 6NR Caregiver Reflections on Vitamais** |
| Q504. | So what are the kinds of things that mothers and fathers can do to improve their child’s health?  *If caregiver says something about “giving good food” or “proper feeding,” probe for what that means.* |  | |
| Q505. | What are the reasons these things help improve a child’s health? |  | |
| Q506. | So we were just talking about keeping children **healthy**, but I’m wondering if there is anything you can ***give*** to help your child ***grow***?  *Can say “anything the child could eat, drink, swallow or receive into their body,” if caregiver needs an example.*  *Probe for why these things are good for child growth.*  *Make a note about whether she specifically mentions “vitamins,” or whether she says something else to refer to Vitamais’ contents, such as “things it contains,” “medicines,” “ingredients,” etc. or other components.* |  | |

| **Module 6NR: Caregiver Reflections on Vitamais** | | |
| --- | --- | --- |
| **MODULE INTRODUCTION: “**You have provided so much helpful information so far, and I really appreciate that. If it’s okay, I have just a few more questions I would like to get your views on before we end with a few questions about your household.” | | |
| Q601. | As you know, there are vouchers for Vitamais only for children who are between 6 and 23 months. I’m wondering if you think it is a problem that there are no vouchers for children once they are two years old? Or is that okay?  *Probe for reasons why caregiver thinks it is or isn’t a problem that Vitamais is only for children 6-23 months old.* |  |
| Q602. | What do others in your household and community think about Vitamais?  *Probe for as many opinions about Vitamais as caregiver can provide.*  *Probe for the reasons for those opinions and if the opinions have changed over time.* |  |
| Q603. | Do you have any final thoughts you would like to share with us that we haven’t already talked about? |  |

| Module 7NR. Socio-demographic Information | | | | |
| --- | --- | --- | --- | --- |
| **MODULE INTRODUCTION:** “Thank you so much for all of the thoughtful information you’ve given me today. It makes a big difference for all of us to understand, first-hand, what mothers’ experiences with the vouchers are as well as with their child’s health. I know this will be very helpful. I just have a few final questions about your household.” | | | | |
|  | **QUESTION** | **ANSWER** | **CATEGORIES AND CODES** | **SKIP** |

| Q701 | How many people, in total, currently live in your household?  HOUSEHOLD IS DEFINED AS "ALL PEOPLE LIVING IN THE HOUSE FOR THE LAST 6 MONTHS AND ATE ON THE SAME POT " | TOTAL |  |
| --- | --- | --- | --- |
|  |  | REFUSES TO ANSWER | 97 |
|  |  | DOES NOT KNOW | 98 |
| Q702 | how many children under 5 years old live in the household?  including the target child | TOTAL NUMBER OF CHILDREN |  |
| Q703 | Of the children living in the household, who is in the age range between 6 to 23 months?  INCLUDING THE TARGETED CHILD, IF APPLICABLE | TOTAL NUMBER OF CHILDREN  IF NONE, '00' |  |
| Q704 | Of the children living in the household, who is in the age range between 24 to 59 months (2 to 5 years old)?  INCLUDING THE TARGETED CHILD, IF APPLICABLE | TOTAL NUMBER OF CHILDREN  IF NONE, '00' |  |
| Q705 | What is the native language of the head of the household? | SENA | 01 |
|  |  | NDAU | 02 |
|  |  | SHONA | 03 |
|  |  | PORTUGUESE | 04 |
|  |  | OTHER (SPECIFY): _________________________ | 96 |
| Q706 | What is your relation to (CHILD NAME)? | MOTHER | 01 |
|  |  | GRANDMOTHER | 02 |
|  |  | BROTHER | 03 |
|  |  | OTHER (SPECIFY): _________________________ | 96 |
| Q707 | How old are you?  INDICATE THE AGE OF THE INTERVIEWEE | AGE |  |
|  |  | REFUSES TO ANSWER | 97 |
|  |  | DOES NOT KNOW | 98 |
| Q708 | Finally, I’d like to know about your schooling. Did you go to school? Please tell me what schooling you have had. | ADULT LITERACY PROGRAM | 01 |
|  |  | PRIMARY EP1 (GRADE 1 TO 5) | 02 |
|  |  | PRIMARY EP2 (GRADE 6 TO 7) | 03 |
|  |  | SECONDARY ESG1 (GRADE 8 TO 10) | 04 |
|  |  | SECONDARY ESG2 (GRADE 11 TO 12) | 05 |
|  |  | ELEMENTARY TECHNICAL EDUCATION | 06 |
|  |  | BASIC TECHNICAL EDUCATION | 07 |
|  |  | MIDDLE TECHNICAL EDUCATION | 08 |
|  |  | TEACHING COURSE | 09 |
|  |  | SUPERIOR | 10 |
|  |  | OTHER (SPECIFY): _______________________ | 96 |
|  |  | DOES NOT KNOW | 98 |

**“This is now the end of the interview. Thank you again so much for your participation and your time. It is so helpful for us to get your perspective and learn more about your experiences as well as those of other mothers, grandmothers and caregivers in the community that we have had or will have the opportunity to talk with. All of the information you have shared today will help us make programs better for you, your children, your family and your community. Thank you!”**
